# Repetitive mild traumatic brain injury-induced neurodegeneration and inflammation is attenuated by acetyl-L-carnitine in a preclinical model

**DOI:** 10.3389/fphar.2023.1254382

**Published:** 2023-09-08

**Authors:** Matthew I. Hiskens, Katy M. Li, Anthony G. Schneiders, Andrew S. Fenning

**Affiliations:** ^1^ Mackay Institute of Research and Innovation, Mackay Hospital and Health Service, Mackay, QLD, Australia; ^2^ School of Health, Medical and Applied Sciences, Central Queensland University, Rockhampton, QLD, Australia

**Keywords:** head injury, concussion, chronic traumatic encephalopathy, CTE, treatment, animal model

## Abstract

Repetitive mild traumatic brain injuries (rmTBI) may contribute to the development of neurodegenerative diseases through secondary injury pathways. Acetyl-L-carnitine (ALC) shows neuroprotection through anti-inflammatory effects and via regulation of neuronal synaptic plasticity by counteracting post-trauma excitotoxicity. This study aimed to investigate mechanisms implicated in the etiology of neurodegeneration in rmTBI mice treated with ALC. Adult male C57BL/6J mice were allocated to sham, rmTBI or ALC + rmTBI groups. 15 rmTBIs were administered across 23 days using a modified weight drop model. Neurological testing and spatial learning and memory assessments via the Morris Water Maze (MWM) were undertaken at 48 h and 3 months. RT-PCR analysis of the cortex and hippocampus was undertaken for MAPT, GFAP, AIF1, GRIA, CCL11, TDP43, and TNF genes. Gene expression in the cortex showed elevated mRNA levels of MAPT, TNF, and GFAP in the rmTBI group that were reduced by ALC treatment. In the hippocampus, mRNA expression was elevated for GRIA1 in the rmTBI group but not the ALC + rmTBI treatment group. ALC treatment showed protective effects against the deficits displayed in neurological testing and MWM assessment observed in the rmTBI group. While brain structures display differential vulnerability to insult as evidenced by location specific postimpact disruption of key genes, this study shows correlative mRNA neurodegeneration and functional impairment that was ameliorated by ALC treatment in several key genes. ALC may mitigate damage inflicted in the various secondary neurodegenerative cascades and contribute to functional protection following rmTBI.

## 1 Introduction

Traumatic Brain Injury (TBI) is a global public health problem, with an estimated 69 million (95% CI 64–74 million) TBIs occurring worldwide each year (Dewan et al., 2018). The leading causes of TBI are falls, wars, transport accidents, and sports (Langlois et al., 2006). Mild TBI (mTBI) accounts for between 75%–90% of total TBI cases (Åhman et al., 2013) and can result in persistent cognitive dysfunction (Silver et al., 2009; Strain et al., 2015). Additionally, it is now recognized that individuals who are exposed to repeated mTBI (rmTBI) have increased susceptibility to the development of neurodegenerative disorders including chronic traumatic encephalopathy (CTE) (Lehman et al., 2012; Nordström et al., 2014; Mez et al., 2017; Montenigro et al., 2017).

TBI pathology involves primary injury which induces secondary injury pathways. The primary injury encompasses the physical damage resulting from external impact transmitted to the brain. In contrast, secondary injury involves concurrent and self-exacerbating molecular and chemical changes that occur over time that amplify the damage caused by the primary injury (Kabadi and Faden, 2014). These secondary injury mechanisms include cellular excitotoxicity, oxidative stress, and dysregulated neuroinflammation by chronic activation of astrocytes and microglia, among others (Abdul-Muneer et al., 2015). Excitotoxicity occurs through the overactivation of glutamate receptors, leading to an influx of calcium ions which can generate further neuronal damage and trigger apoptosis (Morimoto et al., 2002). In addition, this leads to excessive mitochondrial uptake of calcium resulting in oxidative stress (Fehily and Fitzgerald, 2017) and induces other pathological mechanisms including cytoskeletal damage (Deng et al., 2007). Dysregulated inflammation through the chronic release of pro-inflammatory cytokines and chemokines also contributes to the progression of secondary injury (Simon et al., 2017). Understanding these secondary injury pathways is critical for developing effective neuroprotective strategies for TBI, as there are currently no Food and Drug Administration (FDA) approved therapeutic compounds for treatment (Hiskens, 2022).

Acetyl-L-carnitine (ALC) is an endogenously produced carnitine metabolite present in tissue and plasma, and readily crosses the blood brain barrier (BBB) (Jones et al., 2010) unlike its unacetylated form. ALC is also a commonly available nutritional supplement, with a known safety profile, and had been well-studied for the role in aiding β-oxidation of long chain fatty acids in the mitochondria (Bigford and Del Rossi, 2014). ALC demonstrates a wide therapeutic potential in chronic conditions which affect the cardiovascular and nervous system such as diabetes and hypertension (Malaguarnera, 2012). Recently, it has also gained attention as a possible neuroprotective agent, displaying therapeutic potential for neurodegenerative conditions in both pre-clinical and clinical studies (Mancuso et al., 2012). In animal models, ALC has exhibited neuroprotective properties including anti-inflammatory and antioxidant effects (Bogaert et al., 1994; Zanelli et al., 2005; Bagetta et al., 2008; Xu et al., 2015), as well as protection following glutamate-induced neurotoxicity in neurodegenerative states (Nagesh Babu et al., 2011). ALC was able to prevent excitotoxicity-induced N-methyl-D-aspartate (NMDA) reduction in the hippocampus, striatum, and frontal cortex of aged rats (Castorina et al., 1994), regulate synaptic plasticity by counteracting the loss of NMDA receptors in the neuron membrane (Jones et al., 2010), increase the production and activity of neurotrophins (Chiechio et al., 2006), and enhance production of nerve growth factor in brain tissue through increased choline acetyltransferase activity (Piovesan et al., 1994). Studies investigating the therapeutic administration of ALC in rat models of moderate TBI also found beneficial effects. In these studies, ALC administration resulted in smaller lesion volumes, improved sensory and motor function, as well as non-spatial learning compared to controls (Scafidi et al., 2011; Chen et al., 2018).

In human studies, ALC has shown the ability to improve cognitive performance and slow decline in the early stages of neurodegenerative diseases (Spagnoli et al., 1991), as well as reduce neurodegenerative oxidative and nitrosative stress (Calabrese et al., 2003). Taken in conjunction with other nutritional supplements and lifestyle interventions, ALC has also demonstrated cognitive improvements and increased cerebral blood flow in retired American football players with a history of concussion (Amen et al., 2011). Furthermore, a human trial of L-carnitine on patient with severe TBI showed preservation of neurological function after just 1 week of daily administration (Mahmoodpoor et al., 2018). This finding was impressive given the decreased ability of L-carnitine to cross the BBB compared to ALC. While some studies have shown promise for improving clinical and psychometric outcomes in individuals with probable Alzheimer’s disease (AD) and mild cognitive impairment, other studies that included participants with moderate AD progression were less conclusive (Onofrj et al., 2013). It may be that this lack of improvement is related to a therapeutic “window of opportunity”; once the neurodegenerative mechanisms have commenced, a reversal of these processes is not attainable (Bigford and Del Rossi, 2014). Therefore, prophylactic ALC treatment in a paradigm of neurotrauma may be a way to maximize its therapeutic potential.

The translational application of ALC is attractive given that it is an over-the-counter supplement that can be safely administered long-term (Traina, 2016) and is often part of a typical athletic supplement profile. Despite this, there are few studies investigating the therapeutic efficacy of ALC in the context of rmTBI. This study aims to determine whether improvement in genetic markers of inflammation, excitotoxicity, neuronal and glial injury occur as a result of preventative administration of ALC in a mouse model of rmTBI. Measures of neurological function post-injury should also show improvement in learning and motor skill analyses.

## 2 Materials and methods

### 2.1 Animals and general overview

This study was approved by the Animal Ethics Committee of Central Queensland University (CQU AEC 0000021124) in adherence with guidelines from the National Medical Research Council of Australia. The ARRIVE guidelines were used for study design and reporting. A total of 48 male C57BL/6J mice (Animal Resource Centre, Canning Vale, WA, Australia) were used for the study, and were 10 weeks old at the initiation of the protocol. Mice were housed four per cage in a 12:12 h light-darkness cycle, in a constant temperature of 22°C ± 2 °C, with food and water permitted *ad libitum*.

### 2.2 Groups and dosing

**FIGURE 1:**
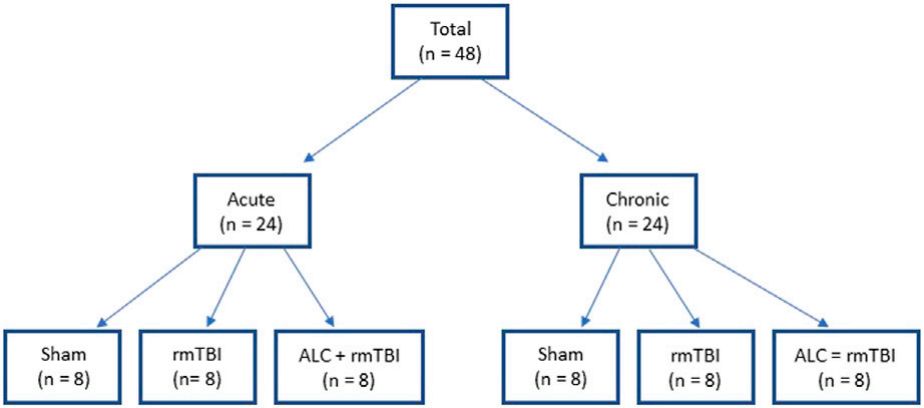
Group allocations for the acute and chronic arms of each treatment.

The study design included two separate arms, acute investigation involving mice euthanized 48 h following final impact, and chronic investigation involving mice euthanized 90 days following final impact (Figure 1). In both the acute and chronic arms, mice were randomized into three groups: Group 1: sham (n = 8), Group 2: rmTBI (n = 8), and Group 3: ALC + rmTBI (n = 8). Animal dosing based on body surface area was undertaken using allometric scaling to reflect the differences in human and mouse metabolic rates. ALC dose was 600 mg kg−1 day−1, based on the FDA allometric scaling factor of 0.081 (Nair and Jacob, 2016). Agents were administered via subcutaneous injection, with the ALC + rmTBI group receiving ALC dissolved in saline, while the sham and rmTBI groups received only saline. Administration commenced 14 days prior to the first mTBI and continued daily until the time of euthanasia of the acute groups. Mice were weighed before ALC administration was commenced, once per week during the mTBI protocol, and at euthanasia. The bodyweight of impacted and non-impacted groups was not significantly different at any time-points (data not shown).

### 2.3 mTBI modelling

**FIGURE 2:**
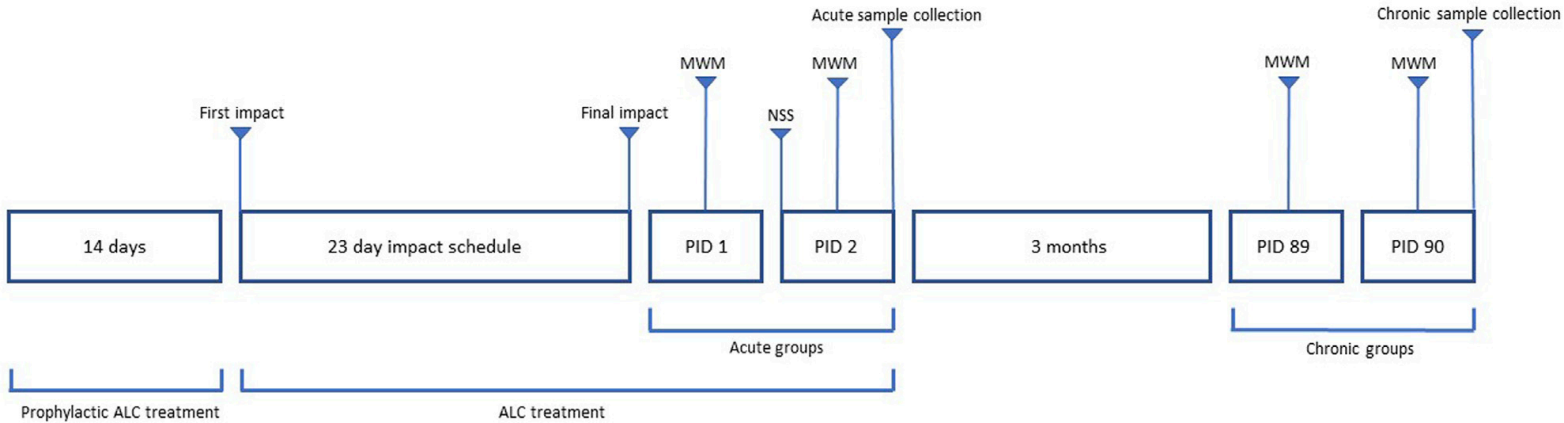
Workflow of dosing, behavior assessment and sample collection. Acute groups had behavioral testing involving MWM and NSS on PID 1 and 2, with sample collection on PID 2. Chronic groups had MWM testing on PID 89 and 90, with sample collection on PID 90.

Mice in the rmTBI and ALC + rmTBI groups undertook 15 rmTBI across 23 days via an apparatus engineered to mimic the head acceleration forces of human mTBI, as described previously (Hiskens et al., 2021a). Prior to impact, anesthetisation of mice was performed using a 1 L inhalation chamber containing 0.5 mL of isoflurane (Zoetis, Rhodes, NSW, Australia) in a cotton ball (yielding a steady 4% concentration). Mice were placed in the chamber until light anaesthesia was indicated by lack of response to tail pinch. Following anesthetisation, the mouse was positioned chest down on the apparatus platform, which consisted of two magnetically adjoined panels designed to collapse upon impact and provide minimal platform resistance. Impacts were applied using a 25 g steel cylinder (12 mm diameter) with a circular rubber impactor (10 mm diameter) attached to the impact surface. The weight was dropped from a height of 1 m and guided through a vertical tube (15 mm diameter) that was aligned above the head. Upon impact, the mouse fell and landed on a padded sponge cushion in a supine position. The impact weight was tethered to restrict the fall of the weight following impact to avoid inadvertent additional contact. Following mTBI the mouse was moved to a recovery heating pad. No mice died during impact or recovery, no mice had evidence of subdural haematoma or skull fracture at post-mortem analysis, and all mice were included in the final analysis. Mice in the sham group received anaesthesia identical to the impact groups but did not receive rmTBI. Figure 2 provides the workflow of dosing, impacts, behaviour assessment and sample collection following final impact.

### 2.4 Neurological and spatial learning assessments

The righting reflex (RR) recovery time was assessed after each mTBI to provide a measure of neurological restoration in all groups. Mice were positioned on their back and the time to adopt a prone position was recorded. The RR time was defined as from the cessation of isoflurane inhalation to the commencement of the RR.

### 2.5 Neurological severity score

Neurological function was assessed using the 10-point neurological severity score (NSS) as undertaken previously (Luh et al., 2011). The NSS involves 10 individual tasks that evaluate motor ability, alertness, balance, and behavior. One point is awarded for each task the mouse is unable to perform as identified in Table 1. NSS was determined 48 h following final rmTBI or sham anesthetization. The NSS tasks were undertaken by an investigator blinded to group allocation.

**TABLE 1:** Tasks of neurological severity score.

Task	Points
Presence of mono- or hemiparesis	1
Inability to walk on a 3-cm-wide beam	1
Inability to walk on a 2-cm-wide beam	1
Inability to walk on a 1-cm-wide beam	1
Inability to balance on a 1-cm-wide beam	1
Inability to balance on a round stick (0.5 cm diameter)	1
Failure to exit a 30-cm-diameter circle (for 2 min)	1
Inability to walk straight line	1
Loss of startle behavior	1
Loss of seeking behavior	1
**Maximum total**	**10**

Bold value is the total score of the test.

### 2.6 Morris Water Maze

Spatial learning and memory were assessed via the Morris Water Maze (MWM) (Vorhees and Williams, 2006), as described by our laboratory previously (Hiskens et al., 2021b). Briefly, a circular 110 cm diameter tank was used, and visual cues were positioned around the tank. The water temperature was 27±1°C, and white, non-toxic paint (Fine Art Supplies, Auckland, NZ) was used to make the water opaque. A hidden circular platform was positioned in the northern quadrant below the surface of the water. Four trials, each consisting of three attempts from different starting quadrants (south, east and west), were administered across 2 days. The trials occurred on post-impact day (PID) 1 and 2 for the acute groups, and PID 89 and 90 for chronic groups (n = 8 per group). For each attempt, mice had a maximum time of 60 s to find and remain on the hidden platform. Mice that did not locate the platform within the allocated time were guided to the platform and allowed to remain on the platform for 10 s. Following the final trial on PID 90, mice in the chronic group also underwent a probe trial, which involved removal of the hidden platform from the pool. Mice commenced in the southern quadrant and were allocated 30 s to search for the platform. Time spent in the northern quadrant was assessed. The investigator conducting MWM assessment was blinded to group allocation. Kinovea 0.8.15 software was used to measure trial times, and to track swim speed as a measure of deficits in motor function.

### 2.7 Sample collection

Euthanasia was performed via isoflurane inhalation until the cessation of vital signs and absence of pedal reflex. The brain was removed, weighed, and washed in ice cold oxygenated (95% O 2, 5% CO 2) artificial cerebrospinal fluid (CSF) containing 118.0 mM NaCl, 3.5 mM KCl, 1.3 mM MgCl2, 26.2 mM NaHCO3, 1.0 mM NaH2PO4, 2.5 mM CaCl2, 11.0 mM glucose. The cortex and hippocampus tissues were then dissected on a frozen dissection platform, and frozen at −80°C for genetic analysis. Collection tubes were coded to enable blinding of the molecular analysis.

### 2.8 RT-PCR

The following gene expression changes were assessed - MAPT (microtubule associated protein tau), GFAP (glial fibrillary acidic protein), AIF1 (allograft Inflammatory Factor 1), GRIA1 (glutamate ionotropic receptor AMPA type subunit 1), TNF (tumor necrosis factor), CCL11 (C-C motif chemokine 11), and TDP-43 (TAR DNA-binding protein 43). mRNA was extracted from tissue homogenates of the hippocampus and cerebral cortex of rmTBI and control groups (n = 4 per group at 48 h or 90 days after injury) using the phenol-chloroform method (Sambrook and Russell, 2006). Sample concentration and purity were evaluated using a spectrophotometer (NanoDrop 2000c). Complementary DNA was synthesized using Superscript III First-Strand Synthesis System for reverse transcriptase-PCR according to the manufacturer’s instructions (Applied Biosystems, Foster City, CA, United States) and run in a thermal cycler (T100 Thermal Cycler, Bio-Rad, Gladesville, NSW, Australia). Samples and negative controls were prepared in duplicate using Taqman universal PCR master mix and run using a thermal cycler (RotorGene Q, Qiagen, Venlo, Netherlands). The following Taqman gene expression assays were used (Applied Biosystems catalogue numbers): mouse MAPT (Mm00521990_m1), GFAP (Mm01253030_m1), AIF1 (Mm00479862_g1), GRIA1 (Mm00433753_m1), TNF (Mm00443258_m1), CCL11 (Mm00441238_m1), TARDBP (Mm01257504_g1), and gene products were normalized to endogenous mouse GAPDH (Mm99999915_g1). Relative expression for Taqman analyzed transcripts was calculated using the delta-delta Ct method (Livak and Schmittgen, 2001).

### 2.9 Statistical analyses

An *a priori* power analysis used α of 0.05, power of 0.8, and means and SD from previous laboratory data, and determined that eight mice per group were required for cognitive and neurological tests. Statistical analyses were performed using IBM SPSS Statistics for Windows Version 25.0 (IBM Corp, Armonk, NY). Data were evaluated for normality via Shapiro-Wilks test prior to formal testing. One-way ANOVA, or repeated-measures two-way ANOVA, with Tukey *post hoc* tests (alpha <0.05) was used to assess for statistically significant differences. All data are presented as means with standard deviations.

## 3 Results

Mice in the rmTBI and the ALC + rmTBI groups showed no signs of physical stress following impacts, and no mice were withdrawn from the study. Mice demonstrated no adverse effects from the administration of ALC.

### 3.1 Righting reflex

**FIGURE 3:**
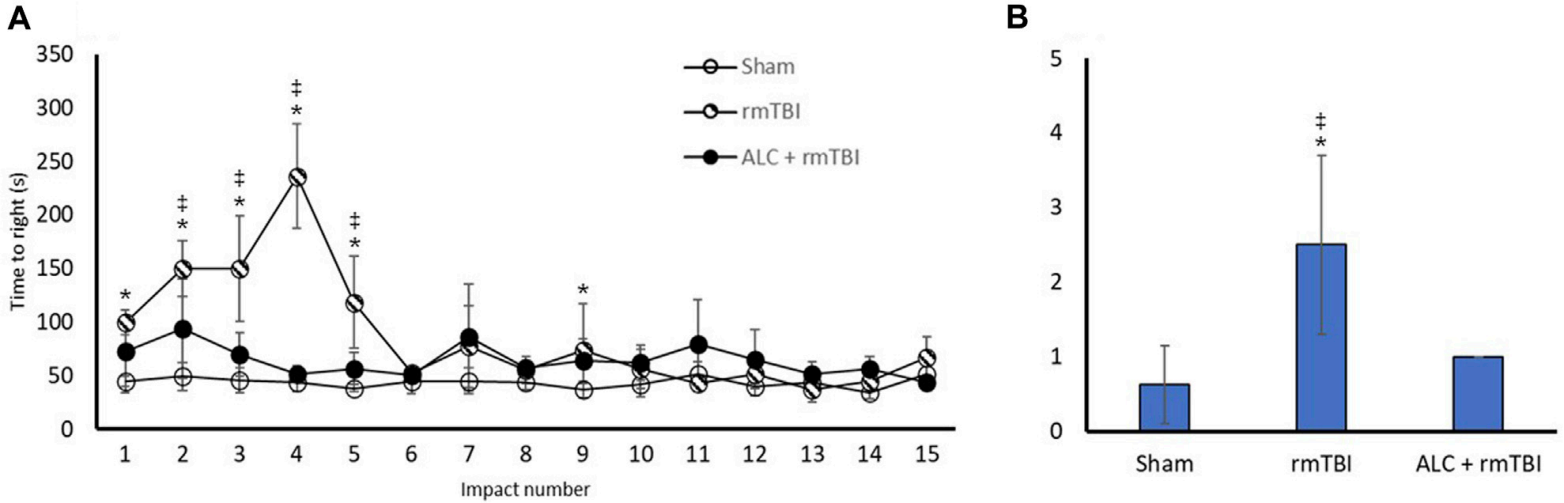
Recovery of righting reflex and neurological severity score following rmTBI and acetyl L-carnitine treatment. **(A)** Time to regain righting reflex (seconds) following impact or sham control anesthesia. **(B)** Values reported as mean (±SD). *p < .05 difference compared with Sham; ‡*p* < .05 difference compared with ALC + rmTBI. N = 8 per group.

Recovery of RR was measured following all impacts or Sham group anesthetizations (Figure 3A). The rmTBI group had delayed RR compared with the Sham group following impacts one through five and impact nine (*p* < 0.05). For all other impacts there was no difference between rmTBI and Sham groups. There were significant differences in RR time between the rmTBI and ALC + rmTBI groups following impacts two through five (*p* < 0.05), with no difference between these groups following impacts six through 15. There were no significant differences between the Sham and ALC + rmTBI group following any impact.

### 3.2 NSS

NSS was undertaken in the acute phase of recovery (Figure 3B). The rmTBI group had significantly greater scores than the Sham or ALC + rmTBI groups (*p* < 0.05). The ALC + rmTBI group score was not significantly different from Sham group.

### 3.3 Spatial learning and memory

**FIGURE 4:**
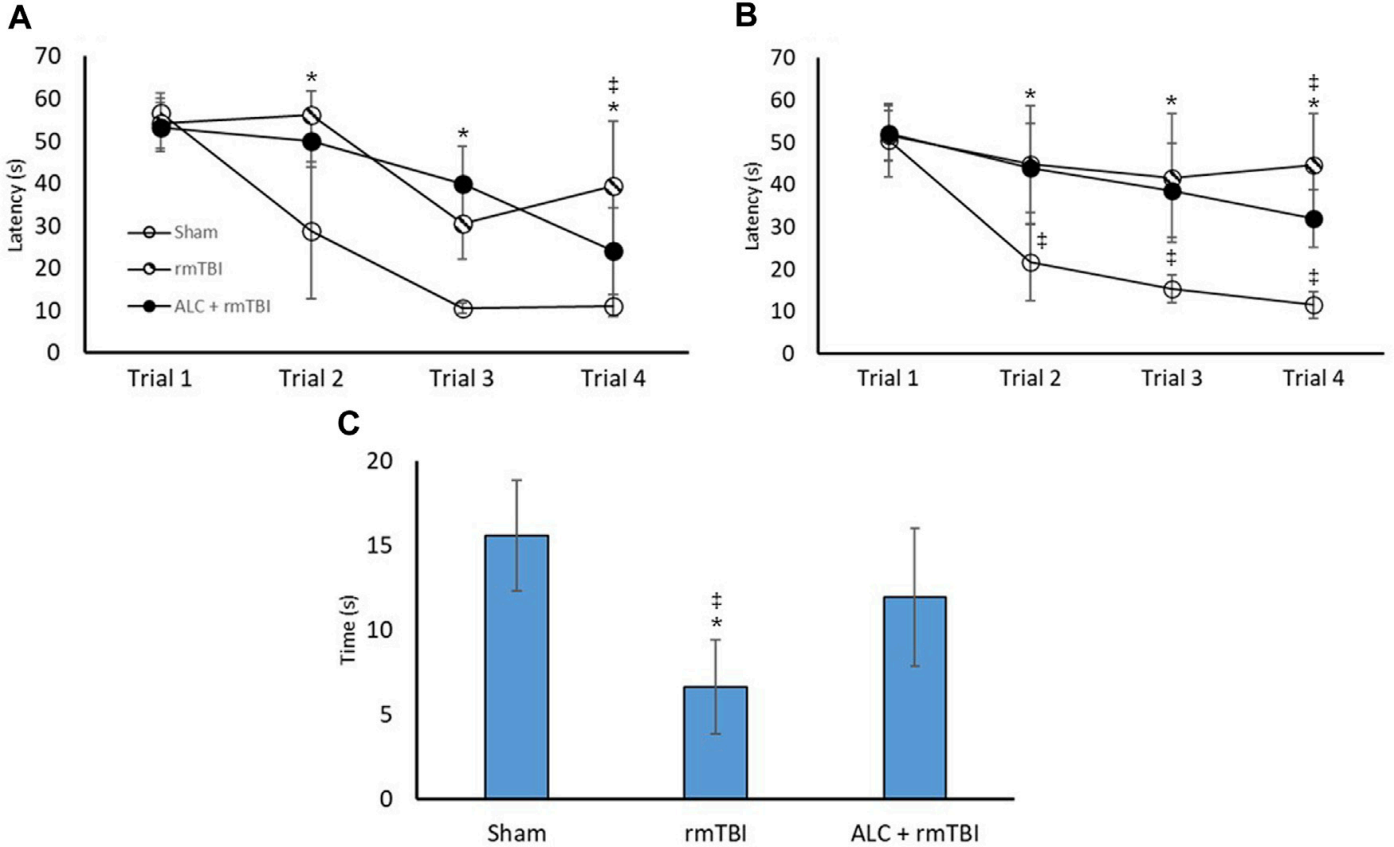
Repetitive mTBI and acetyl L-carnitine treatment influences performance in the Morris Water Maze. **(A)** Time to find the hidden platform (seconds) in the MWM at acute testing. **(B)** Time to find the hidden platform in the MWM at chronic testing. **(C)** Time spent in the goal quadrant of the Probe Test at chronic testing. Values reported as mean (±SD). *p < .05 difference compared with Sham; ‡*p* < .05 difference compared with ALC + rmTBI. N = 8 per group.

Figure 4 demonstrates the MWM assessment 48 h and 3 months following final injury. In the acute testing period, latency to find the hidden platform was significantly different between the Sham group and the rmTBI group at trials 2, 3, and 4 (*p* < .05). Notably, there was a significant difference between rmTBI and ALC + rmTBI group times in trial 4 (*p* < .05). There were no significant differences in trial times between the Sham and ALC + rmTBI groups. In the 3-month testing period, there were group differences between the Sham and rmTBI groups at trial 2, 3, and 4 (*p* < .05). At trial 4, mean group times were again significantly different between the rmTBI group and the ALC + rmTBI groups (*p* < .05). There were significant differences between the Sham and ALC + rmTBI groups at trial 2, 3, and 4 (*p* < .05). Swim speed was not different between any of the groups at any of the trials, which indicates that differences seen in time to find the platform were related to cognitive deficits rather than motor impairment.

Probe trials were undertaken in the chronic testing arms, with the rmTBI displaying impaired searching ability compared with the Sham and the ALC + rmTBI groups (*p* < .05). There was not a significant difference between the Sham and the ALC + rmTBI groups.

### 3.4 RT-PCR

**FIGURE 5:**
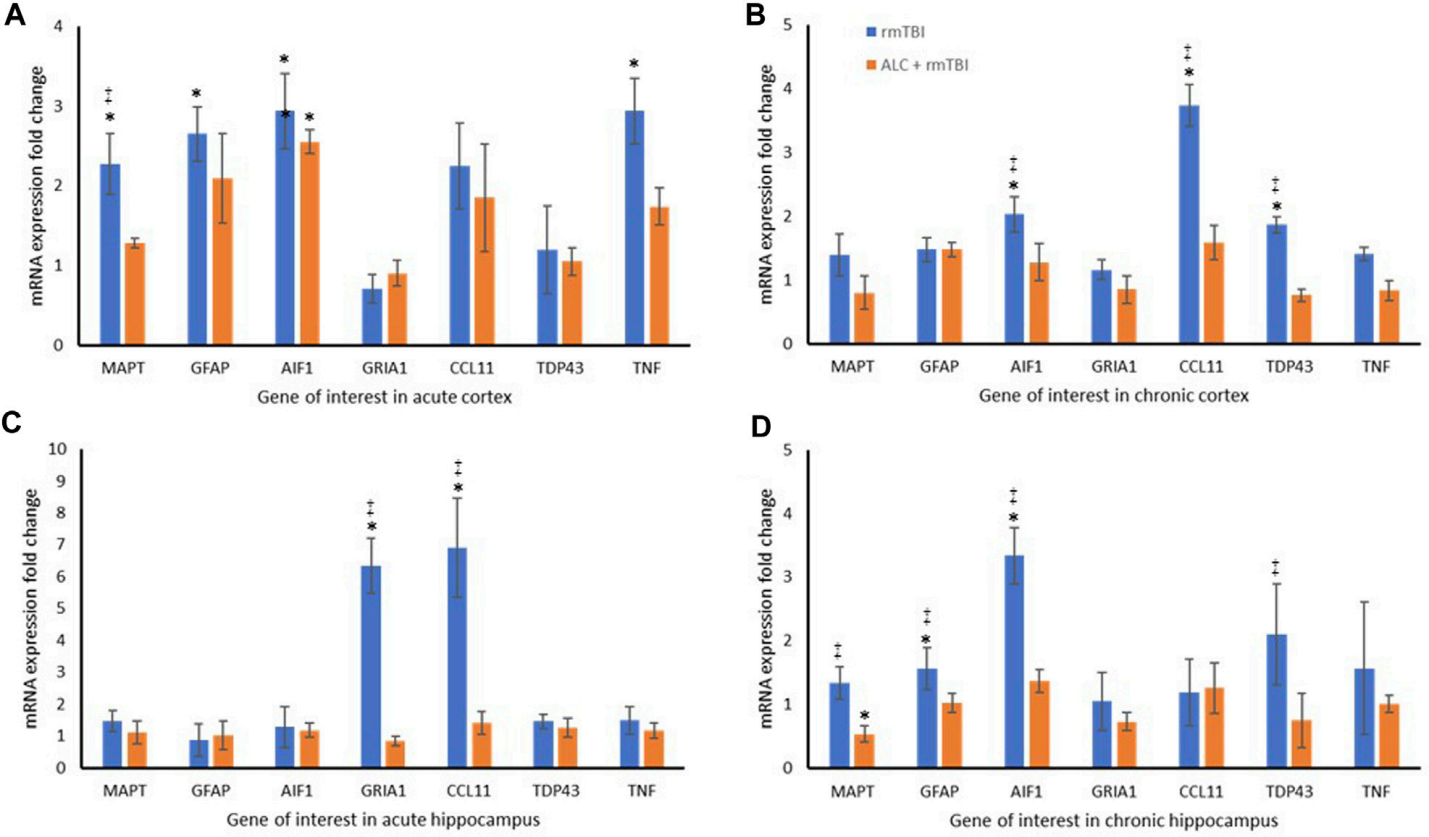
Repetitive mTBI and ALC treatment influences expression change of neurodegenerative genes. **(A)** mRNA expression fold change relative to sham control of eight genes in the cortex at acute testing. **(B)** mRNA expression fold change relative to sham control of eight genes in the cortex at chronic testing. **(C)** mRNA expression fold change relative to sham control of eight genes in the hippocampus at acute testing. **(D)** mRNA expression fold change relative to sham control of eight genes in the hippocampus at chronic testing. Normalized to Gapdh (±SD). *p < .05 difference compared with Sham; ‡*p* < .05 difference compared with ALC + rmTBI. N = 4 per group.

The molecular analysis assessed expression changes between the three groups for seven genes, with differential expression seen between the cortex and hippocampus. At acute injury assessment in the cortex, MAPT, GFAP, AIF1 and TNF showed upregulation in the rmTBI group relative to Sham mice (*p* < .05). Assessment of acute cortex also revealed significant differences between the rmTBI and ALC + rmTBI groups in MAPT expression (*p* < .05), and group differences between the sham and ALC + rmTBI group in AIF1 expression (Figure 5A). In the cortex from the chronic treatment arm animals, the rmTBI group were significantly different to Sham and ALC + rmTBI groups for AIF1, CCL11, and TDP43 expression (*p* < .05) (Figure 5B). In acute hippocampus tissues, the rmTBI groups showed differential expression to Sham and ALC + rmTBI groups in GRIA1 and CCL11 genes (*p* < .05) (Figure 5C). In the hippocampus from the chronic treatment arms, rmTBI groups were significantly different to Sham and ALC + rmTBI groups for GFAP and AIF1 genes (*p* < .05) (Figure 5D). The rmTBI group showed significant differences to the ALC + rmTBI for MAPT and TDP43 expression (*p* < .05). All gene expression data are shown in Tables 2, 3.

**TABLE 2:** Statistical results obtained from the one-way ANOVAs for changes in expression of the seven genes of interest for the two brain regions at the acute sacrifice time (48 h post-mTBI) (significant differences *p* < .05 are in bold).

Cortex			Post-hoc		
	F	P	Sham: rmTBI	Sham: ALC + rmTBI	rmTBI: ALC + rmTBI
MAPT	17.66	**<.01**	**<.01**	.31	**<.01**
GFAP	5.80	**.01**	**<.01**	.05	.76
AIF1	6.90	**<.01**	**<.01**	**.02**	.95
GRIA1	3.58	.05	-	-	-
CCL11	3.21	.06	-	-	-
TARDBP	3.37	.06	-	-	-
TNF	10.27	**<.01**	**<.01**	.05	.09
Hippocampus					
MAPT	4.76	**.02**	.06	.83	.23
GFAP	0.99	.43	-	-	-
AIF1	0.65	.60	-	-	-
GRIA1	23.01	**<.01**	**<.01**	.92	**<.01**
CCL11	7.32	**<.01**	**<.01**	.85	**.02**
TARDBP	5.68	**.01**	.55	.82	.95
TNF	0.88	.48	-	-	-

**TABLE 3:** Statistical results obtained from the one-way ANOVAs for changes in expression of the seven genes of interest for the two brain regions at the chronic sacrifice time (3 months post-mTBI) (significant differences *p* < .05 are in bold).

Cortex			Post-hoc		
	F	P	Sham: rmTBI	Sham: ALC + rmTBI	rmTBI: ALC + rmTBI
MAPT	1.90	.18	-	-	-
GFAP	2.56	.10	-	-	-
AIF1	10.69	**<.01**	**<.01**	.36	**.04**
GRIA1	3.70	**.04**	.45	.39	**.03**
CCL11	8.97	**<.01**	**<.01**	.32	**.03**
TARDBP	43.90	**<.01**	**<.01**	.04	**<.01**
TNF	2.45	.11	-	-	-
Hippocampus					
MAPT	29.57	**<.01**	.08	**<.01**	**<.01**
GFAP	7.15	**<.01**	**.01**	1	**.02**
AIF1	22.72	**<.01**	**<.01**	.21	**<.01**
GRIA1	3.07	.07	-	-	-
CCL11	0.81	.51	-	-	-
TARDBP	7.13	**<.01**	.08	.75	**.01**
TNF	1.72	.22	-	-	-

## 4 Discussion

There is currently a lack of evidence for safe therapeutics that can be administered long-term to reduce the risk of individuals developing cognitive and neuropsychological deficits after rmTBIs. This study evaluated the role of prophylactic ALC administration on reducing the secondary injury mechanisms of axonal neurodegeneration, reactive astrogliosis and microgliosis, inflammation and glutamate excitotoxicity in the hippocampus and cortex resulting from rmTBI. The dosing profile followed a modified prevention paradigm to fully supplement the anti-inflammatory and metabolic cellular protective pathways targeted by ALC supplementation. The mTBI model in this study has been characterized to deliver repetitive, biomechanically relevant impacts, to induce CTE-like neuropathology and cognitive impairment (Hiskens et al., 2021c). Through the evaluation of TDP43, MAPT, AIF1, GFAP, TNF, CCL11 and GRIA mRNA gene expression in the cortex and hippocampus, we show that ALC mitigated damage inflicted in these secondary neurodegenerative cascades. This was also confirmed by improvements in behavioral and cognitive function.

TDP-43 is an important protein for stabilization of RNA processes such as transcription, mRNA stability and alternative splicing (Jo et al., 2020). Recent discoveries have implicated the protein in several neurodegenerative diseases such as Alzheimer’s disease, Parkinson disease, amyotrophic lateral sclerosis, Lewy body dementia and frontotemporal dementia and CTE (Jaiswal et al., 2022). Thus, TPD-43 was an important marker to confirm the presence of chronic neurodegeneration in our study. While our results indicated that the TDP-43 gene expression showed no significant changes in the acute phase in both the cortex and hippocampus, within the chronic arm (at 3 months), TDP-43 was significantly elevated in the impact group relative to the ALC + rmTBI treatment groups in both the cortex and the hippocampus. This is consistent with TDP-43 protein aggregation being a key feature of chronic neurodegenerative conditions (McKee et al., 2016), but rarely seen in the acute phase of injury or disease (McKee et al., 2010). It is believed its aggregation in the cytoplasm is mostly due to factors such as mislocation of the protein from the nucleus in states of cellular stress, as well as defective clearance mechanisms from the cytoplasm (Meneses et al., 2021). Incorrect TDP-43 signaling induced by mTBI damage may also contribute to the impaired transport of proteins and organelles within neurons, leading to the aggregation of axonal debris that contributes to neurofibrillary tangles (NFTs) commonly found in CTE (Cherry et al., 2016).

A key constituent of these NFTs is tau, a microtubule-associated protein involved in axon construction (Moreno-Gonz et al., 2011). In our study, we observed upregulation of MAPT (microtubule associated protein tau) mRNA levels in the cortex of the rmTBI group at 48 h, which was significantly different from the non-impacted sham and ALC + rmTBI groups. Although MAPT expression change was less pronounced in the cortex of the rmTBI group at 3 months, significant differences were observed compared to the ALC-treated impact group, suggesting a neuroprotective mechanism that may preserve tau function and organization within the axon. ALC has been found to have protective effects in other tau-dependent neurodegenerative disease (Yin et al., 2010), but the tau-mediating involvement of ALC in mTBI has not been previously demonstrated.

In the acute phase following rmTBI, microglia activate inflammation as a key secondary injury response, aimed at providing immediate neuroprotection. The response induces cytokine production, arachidonic acid conversion to prostaglandins, and excitotoxin release, intended to repair damage and return homeostasis (Corps et al., 2015). However, repetitive or severe trauma brings prolonged microglial activation and chronic inflammation, damaging neurons and glia and ultimately inducing neurodegeneration (De Beaumont et al., 2009; Loane and Kumar, 2016). ALC has been shown to reduce microglial activation and protect against neuroinflammation in a Parkinson’s Disease model (Singh et al., 2018; Burks et al., 2019). In the present study microglial activation was assessed by allograft inflammatory factor 1 (AIF1) gene expression. Following rmTBI, AIF1 significantly increased in the cortex at both 48 h and 3 months, and in the hippocampus at 3 months, indicating the increased inflammation and microgliosis typically observed following TBI. As proposed, ALC administration showed significant attenuation of AIF1 in both tissues in the chronic phase compared to the rmTBI group in this study. This is an interesting distinction, since microglia are known to shift towards destructive functions in the chronic phase of injury, while the acute response may deliver beneficial pro-regenerative and neuroprotective substrates (Loane and Kumar, 2016).

Astroglial cells provide another mechanism for inflammation after mTBI, as they communicate with microglia to respond to the demands of the central nervous system (CNS) (Loane and Kumar, 2016). Glial fibrillary associated protein (GFAP) is an established indicator of astrocyte activity in human head trauma, and in this study an increase in GFAP mRNA expression was observed in the impact group in acute cortex and chronic hippocampus tissues, consistent with previous animal mTBI studies (Mychasiuk et al., 2016). In the ALC + rmTBI groups, the effects of impact were modulated by ALC, with levels not significantly different from the non-impacted control group. This data indicates that ALC can reverse the astrocytosis in the hippocampus that ensues following rmTBI. This effect is consistent with previous findings showing decreased astrocytic reactivity following ALC administration in models of neurodegeneration (Burks et al., 2019) and epilepsy (Tashakori-Miyanroudi et al., 2022).

The inflammatory cascade driven by glial cell response to injury involves the release of products such as cytokines, and our study assessed the levels of pro-inflammatory cytokine, TNF. ALC administration has been shown to decrease TNF activation in an epilepsy model (Tashakori-Miyanroudi et al., 2022). In our study, TNF increased in the cortex tissue of mice 48 h following mTBI. In the ALC + rmTBI group there was no difference in TNF mRNA expression compared to the sham control group at this, or any timepoint. TNF plays a critical role in initiating and increasing the mobilization of microglia and astrocytes following CNS injury (Woodcock and Morganti-Kossmann, 2013), and the relationship between TNF, AIF1, and GFAP expression change in response to mTBI and treatment is valuable for further exploration.

We sought further evidence of the neuroinflammatory response to rmTBI and ALC treatment by examining the expression of CCL11, a chemokine that has received increased attention in TBI (Kovac et al., 2011). CCL11 can have neuroreparative mechanisms, however excessive release can lead to oxidative stress and excitotoxic processes that cause synaptic dysfunction and neuronal death (Parajuli et al., 2015). Studies in mice have linked high levels of CCL11 to cognitive and memory impairment (Villeda et al., 2011), while elevated levels in the cortex of former American football players with CTE correlated with tau pathology and the number of years playing football (Cherry et al., 2017). The present study found increased mRNA expression of CCL11 in rmTBI mice in the cortex at chronic measurement, and in the hippocampus at 48 h post-injury. In both conditions, rmTBI groups were also significantly different to ALC + rmTBI treatment groups, whose CCL11 expression was no different to control. Understanding the potential role of CCL11 as a biomarker of mTBI and in chronic neuropathology is an area for future investigation.

Another important secondary injury mechanism following mTBI is excitotoxicity, involving impaired neuronal calcium regulation and glutamate release resulting in dendrite damage (Lewerenz and Maher, 2015). Upregulation of N-methyl-D-aspartic acid (NMDA) GluA1 receptors is triggered to counteract the damage caused by glutamate build-up in the synaptic cleft, indicated by GRIA1 mRNA expression. The effect of ALC in correcting dysfunctional glutamatergic neurotransmission has previously been demonstrated in a model of depression (Nasca et al., 2013). The hippocampus is especially vulnerable to excitotoxic insult (S et al., 2008), and in the present study rmTBI mice displayed significant hippocampal excitotoxicity at 48 h, with increased GRIA1 expression indicating the attempts to clear excess glutamate. Conversely, ALC + rmTBI mice had significantly lower GRIA1 levels than rmTBI mice and were more similar to those expressed in sham control. This GRIA1 dysregulation may be a contributor to reduced functional outcomes, as spatial working memory relies on the activation GluA1 receptors in the hippocampus (Freudenberg et al., 2013).

We further investigated whether the neuroprotective effects of ALC at the molecular level would translate into mitigation of behavioral and cognitive dysfunction seen following rmTBI. Time to regain RR in the rmTBI group were within the range of previous studies (Dewitt et al., 2013). Importantly, ALC was able to negate the rmTBI-induced RR dysfunction to levels seen in the sham group. Similarly, when undertaking NSS assessment to determine motor, sensory, coordination, and reflex function, rmTBI mice scored poorly while mice treated with ALC performed to the same level as uninjured controls. These data suggest that while the mild impacts resulted in no macroscopic pathology, there was still measurable neurological perturbation in the untreated group that was alleviated by ALC pre-treatment. These interactions may be related to the secondary injury pathways that were assessed via gene expression changes, however further mechanistic studies are required to investigate causation of this effect. Secondary injury to the hippocampus leads to impaired function, which we assessed using the MWM task, to include elements of learning and memory. The rmTBI group displayed impaired performance in the MWM in the acute and chronic testing, indicating the presence of spatial learning and memory deficits, which is consistent with findings from other studies in mice receiving a mTBI (Meehan et al., 2012; Tabet et al., 2022). This test provides an insight into cognitive performance following impact that is relevant in modelling the detriment seen in clinical studies. Evidence from populations such as retired professional football players indicates that repetitive concussive and subconcussive impacts may have long-term implications for cognitive and executive functions, such as working memory and decision making (Hart et al., 2013). At both acute and chronic measurement in our study, treatment with ALC showed protection in later testing sessions, indicating that ALC may contribute to the alleviation of spatial learning deficits that result from rmTBI. Our findings agree with Scafidi and others, who found ALC improved cognition and motor function following moderate TBI (Scafidi et al., 2011). In other studies of neurodegeneration, supplementation with ALC has been found to improve attention, learning and spatial working memory deficits induced by hypoxia (Meneses et al., 2021) and improved MWM performance in a model of Parkinson’s Disease (Singh et al., 2018). ALC has contributed to enhanced learning capacity and cognitive function in rats by providing acetyl groups for acetylcholine synthesis, thereby exerting an effect in cholinergic neurotransmission (Ando et al., 2001). Taken together, these data demonstrate the potential of ALC in eliciting behavioral benefit in the context of rmTBI, and future studies should investigate mechanistic determinants of this effect.

### 4.1 Limitations

Small animal models provide advantages in the study of TBI in the ability to screen for pathology that is otherwise hard to determine. The knowledge that is gained from the findings in mice provide the stimulus to explore the concepts in higher mammals and humans. However, there are limitations to mouse models including the small size and lack of complexity of the mouse brain (Hiskens et al., 2019). This study was limited in that it did not evaluate changes in protein expression in addition to assessing molecular markers. Moreover, it is a limitation of this work that histological sections were not evaluated from the cortex and hippocampus. Additionally, this study only involved male animals, however the investigation of sex differences is recommended for future pre-clinical studies.

## Data Availability

The raw data supporting the conclusions of this article will be made available by the authors, without undue reservation.
